# The Poor Man's Christmas

**Published:** 1890-12-20

**Authors:** 


### The Hospital

A Weekly Journal of

Science, Medicine, Nursing, and Philanthropy.

*For Hospitals, Asylums, Infirmaries, Dispensaries, Medical Practitioners, Students, Nurses, and the Charitable Public.*

Vol. IX.—No. 221.] [December 20, 1890.

### THE POOR MAN'S CHRISTMAS.

"At Christmas play and make good cheer, for Christmas comes but once a year." That was all very well for Master Thomas Tusser, and the jolly farmers to whom he addressed it. The farmer, if he be anything of a man, can right well afford both to play and to make good cheer at Christmas. It often happens, indeed, that nature compels him to play; for she either hides his fields from sight under a mantle of snow, or drenches the soil with cold and dripping rain, or chains it fast in the iron bondage of relentless frost. Under such circumstances there is little to do in the fields, and not much, except the feeding of the cattle, at the homestead. The farmer, too, can make Christmas both merry and plentiful without putting his hand too deeply into his pocket. If his wife be the buxum, rosy-cheeked, and practical dame she ought to be, she has her turkeys in the primest condition and her geese at the fattest at Christmastide. Her well-filled store-room is supplied with the whitest of flour, the richest of butter, and the thickest of cream. Nothing is wanting for the mince pies and the plum puddings but a few sultanas, a little peel, the indispensable almonds, and a modicum of inexpensive seasoning. The holly and the mistletoe, which make Christmas in every room, grow in the coppice and the hedgerows, and can be secured in profusion by the smallest boy on the farm with an hour's labour. Yule logs by the dozen lie about the yard and the out-buildings, almost asking to be offered up as a burnt offering to the Jolly Spirit of the Festive Season. No class of persons in this country can more easily and abundantly surround themselves with the signs of Christmas brightness and mirth than the solid and substantial yeomen of our much-loved England.

That is one side of an annual picture. There is another: but we have a reluctance which we cannot overcome, to the painting of scenes of misery, and the recounting of stories of distress at a time when cares should be banished and lightness of heart should be the order of the day. It is not the poor man's story from the beginning of the year to the end of the year we wish to speak of, but his Christmas; and not his Christmas as it too often is in reality, but his Christmas as it ought to be. For one day in the year, for one week in the year if possible, we earnestly wish that every man, woman, and child in our civilised, scientific, wealthy, and Christian country could be without a care, without a sorrow, without a pain. That, of course, is not possible; but it is the ideal we might all set before ourselves, and which every one of us might do his utmost to bring to actuality and present realisation. Of one thing we are well assured, that the rich and the prosperous are willing to do their share towards removing the heavy load of care and poverty from the poor man's shoulders, and to furnish him with the wherewithal to be careless and joyous for at least a brief period. It can only be for a brief period; for nature does not permit one man to carry another's burden for long, but impartially insists that every man shall manfully shoulder, and sturdily carry his own. But on this very ground—that through the chills of spring, the heats of summer, and the biting frosts of winter, every man is called upon by nature to fight his own daily battle in life—on this very ground is it all the more incumbent upon us when a halt is called, to exchange sympathies with one another, to carry one another's loads for a time, and generally to remember, and to act as if we remembered that "God hath made of one blood all nations of men that dwell upon the face of the earth."

Christmas is the season of giving and taking. The husband gives to the wife, the brother to the sister, the father to the child, and all to one another; and we do not know that any of those receivers of Christmas gifts are pauperised or injured thereby. Is there any good reason why the poor who receive Christmas beef, and pudding, and tea, and even money from their more prosperous neighbours, should be pauperised and injured by such gifts? Is there not a sense in which the rich man is the poor man's brother, and the exalted man is the father of his dependents? Time was, and not so very long ago, when the lord of the soil acknowledged the justice of the poor man's claims, and relieved his necessities without hurt to his self respect. Time was, we have said; but the claims of the poor are as generously recognised by the best men and women of wealth to-day as they have ever been in any past times. There is, it must be admitted, a critical spirit abroad, and sometimes a cynical. We are told to beware of pauperising the industrial population. That is well, within limits. But the apostles of criticism and of the suppression of pauperism have a very decided capacity for the magnifying of their office. They would convert the State into a vast mosaic if they could, and would insist, either upon forcing every man exactly into his own little space, or of crushing him into the smallest of powder with an economical steam-hammer. Now, that is right and good to a certain limited extent. But a living, breathing, thinking, and passionate world can never form a dead and mechanical mosaic. Let us rather aim at making it a well-drilled army, with every soldier in his place, and marching steadily forward at the word of command. But if the world be a well-drilled army, it must, nevertheless, sometimes halt; it must put off its uniform; it must set up its nightly tent, and light its watch-fire; it must have its periods of complete freedom from restraint, of the banishment of care, of boyish playfulness, and of recreation both for body and soul.

Christmas we claim as a halting time for the poor man who has had to trudge so weary a march for the past twelve months; and it ought to be made a joyous time for him. The strict Puritan insists upon the religious character of the Christmas festival. But what is religion? And above all what is the Christian religion? Is it a life that knows no joy; no complete self-abandonment to unrestrained and innocent mirth? If it be it can have no basis in the eternal verities, and must gradually vanish away before the approach of science, like mountain mists before the summer sun as it rises from the Eastern sea. The deeply religious man will spend his Christmas in a deeply religious way, and he will do right. The man of feebler religious instincts will be satisfied with fewer acts of worship and will ask for more physical pleasures, a freer relaxation of the mind. But there are many to whom religion is a comparatively insignificant thing, who yet can enjoy the pleasure of hunger satisfied, of cold banished, of spirits elevated, of family re-union, of drudgery put aside and forgotten for a short space. Are these to have no Christmas joy because their pleasures are different from those of the man of religion? Has Christianity no gifts but those of a spiritual kind? and even those does she dispense only to a strictly chosen few. If we read the New Testament aright the care of Christianity for the bodies of men is limited only by the necessities of those bodies. She bids her apostles and evangelists go into the highways and hedges and compel the poor to come in to her spiritual feasts; and just as much does she bid her apostles and evangelists go into the highways and hedges and compel the needy to come in to her material abundance. Certain fanatics are never happy but when they are pronouncing a divorce between the soul and the body. But the Christianity of the New Testament knows nothing of any such divorce, neither does the science of modern times. The spirit of Christmas urges that all people are to be made happy for a time, and if they have not the capacity for religious enjoyment, let them have such innocent and harmless pleasure as they have the capacity for. The poor have pleasure in sitting down to an abundant table; and so would the rich if they had the spare table of the poor all the year round. Let the poor, then, for one day in the year have a table that will gladden their hearts! They have pleasure in little adornments, in a few evergreens and brightly coloured berries in their homes, in innocent games and family reunions, in homely music and song and dance. Then let them have the means of obtaining all these things, and let the rich Christian strive to expand his heart to the full dimensions of his faith, and give the means of pleasure as God gives the sun and rain, alike to the evil and to the good, to the just and to the unjust. This article will be in the hands of readers some little time before Christmas Day; and we venture to urge that they will put aside all dread of pauperising the poor, and will remember them bountifully wherever they may be placed, whether in their homes, in hospitals, in workhouses, or elsewhere. The season is a season of rejoicing; and it cannot be in the highest sense a period of happiness to any unless it is at the same time a period of happiness to all.

